# It’s a wrap: deriving distinct discoveries with FDR control after a GWAS pipeline

**DOI:** 10.1101/2025.06.05.658138

**Published:** 2025-06-08

**Authors:** Benjamin B. Chu, Zihuai He, Chiara Sabatti

**Affiliations:** 1 Department of Biomedical Data Science, Stanford School of Medicine; 2 Department of Neurology and Neurological Sciences, Stanford School of Medicine; 3 Quantitative Sciences Unit, Department of Medicine, Stanford School of Medicine; 4 Department of Statistics, Stanford University

**Keywords:** GWAS, knockoffs, summary statistics, FDR, data-sharing, UK-biobank

## Abstract

Recent work has shown how to test conditional independence hypotheses between an outcome of interest and a large number of explanatory variables with false discovery rate control (FDR), even without access to individual level data. In the case of genome-wide association studies (GWAS) specifically, summary statistics resulting from the standard analysis pipeline can be used as input of a procedure which identifies distinct signals across the genome with FDR control. This secondary analysis requires sampling of negative controls (knockoff) from a distribution determined by the linkage disequilibrium patterns in the genome of the population under study. In prior work, we have pre-computed this distribution for European genomes, starting from information derived from the UK Biobank. Thus, researchers working with European GWASes can carry out a knockoff analysis with minimal computational costs, using the distributed routine GhostKnockoffGWAS.

Here we introduce and release a new software (solveblock) that extends this capability to a much richer collection of studies. Given a set of genotyped samples, or a reference dataset, our pipeline efficiently estimates the high-dimensional correlation matrices that describe correlation structures across the genome, making rather common sparsity assumptions. Taking this sample-specific estimate as input, the software identifies groups of genetic variants that are highly correlated, and uses them to define an appropriate resolution for conditional independence hypotheses. Finally, we compute the distribution for the exchangeable negative controls necessary to test these hypotheses. The output of solveblock can be passed directly to GhostKnockoffGWAS, allowing users to carry out the complete analysis in a two step procedure.

We illustrate the performance of the routine analyzing data from five UK Biobank sub-populations. In simulations, our method controls FDR. Analyzing real data relative to 26 phenotypes of varying polygenicity in British individuals, we make an average of ≈ 19 additional discoveries, compared to standard marginal association testing. Our code, precompiled software, and processed files for these five subpopulations are openly shared.

## 1 Introduction

The knockoff filter [1] is a variable selection framework that tests conditional independence hypotheses while controlling the false discovery rate (FDR) [2]. The approach is particularly appealing in genome-wide association studies (GWAS), where it leads to interpretable discoveries, that do not require post-processing and are closely tracking causal variants [3]. A successful implementation of this strategy models the distribution of genotypes using hidden-Markov models (HMM) [4, 5], in line with the genetic literature. Prior to a knockoff analysis, however, this approach requires phasing the genotypes, estimating parameters of an HMM, and determining identical-by-descent segments. All these steps are computationally intensive, despite recent advances [6, 7, 8], and require reliance on a collection of external tools not optimized for this specific goal. As a result, a knockoff analysis of GWAS data based on HMMs is still much more involved than the traditional GWAS pipeline.

An alternative path to testing conditional hypotheses across the genome has started to emerge with the realization that an approximate knockoffs analysis can be carried out working not with individual level data, but with summary statistics capturing the correlation between the outcome and the explanatory variables [9, 10]. This procedure, known as GhostKnockoffs, takes as input GWAS summary statistics (e.g. p-value and direction of effect) and can therefore be run without changing any of the standard pre-processing steps. Empirical studies [11] show a particularly appealing characteristic of this pipeline: the input can be any testing statistics that is appropriate for the data, such as those derived from linear mixed models that account for relatedness, as long as its null distribution is *N*(0, 1). In [12] the re-analyses of 60+ European GWASes with this framework lead to greater power and improved precision, as compared with the results of the original analyses.

While the GhostKnockoffs approach has the potential of a more straightforward harmonization with the standard GWAS pipeline, the necessity of modeling the distribution of genotypes and calculating the derived knockoff distribution remains. Since this approach relies on Gaussian approximations, crucial elements are (a) the matrix of correlations between allelic counts of different SNPs and (b) the sampling of powerful second order knockoffs [13]. The authors of [12] distributed a software (GhostKnockoffGWAS) that takes as input GWAS summary statistics and parameters that characterize the knockoff sampling distribution (a,b). To facilitate users, pre-computed parameters appropriate for a European populations derived from LD matrices calculated on data from the UK Biobank [14, 15], were also made available on Zenodo [16].

In this work we intend to provide a broader audience with an easy access to a meaningful and reliable knockoff analysis. Given that GhostKnockoff can be used as a “wrapper” around any standard pipeline, what remains is to provide users with a way of computing the parameters that characterize the knockoff sampling distribution (a,b) that are appropriate for their specific sample. The software we introduce assumes that individual-level genotype data is available. Taking this as input, it (1) estimates high-dimensional LD matrices appropriate for the target sample; (2) it identifies groups of SNPs in high disequilibrium that will be the object of conditional inference; (3) it approximates the dependency structure across these groups; and (4) computes the parameters of the joint variance covariance matrix of the original variables and knockoffs to maximize power.

Thanks to the solveblock binary executable, users can construct the bundle of LD files necessary for GhostKnockoff analysis directly from individual level data stored in VCF [17] or binary PLINK [18] format. To run a genomewide knockoff-based conditional independence testing, then, users need to (i) run any standard GWAS pipeline to obtain marginal Z-scores, (ii) run the solveblock executable to obtain parameters for knockoff construction, and (iii) input the results of these two first steps into GhostKnockoffGWAS. Users have the option (iv) to share the output of solveblock by uploading it to services such as Zenodo [16]. Simulation studies and real-data examples illustrate the speed, enhanced power, and FDR control of knockoff-based inference enabled by this pipeline.

## 2 Methods

We assume that the user has available a set of genotypes X on n individuals (rows) and p SNPs (columns) as well as summary statistics on the association between these same SNPs and a phenotype of interest. The genotypes might have been obtained as part of the study that lead to the association summary statistics, or belong to an appropriate reference panel. We note that when individual level data on genotype and phenotype is available, one can conduct a knockoff-based analysis by generating knockoffs $\tilde{X}$ using HMMs distribution [4, 3, 5]. While there are many robustness advantages of this approach, it remains more computationally involved, and in what follows, we work with a GhostKnockoff [10] pipeline [12].

### Preprocessing steps

For GWAS data with $p\approx 10^{6}$, it is common to assume that the variance-covariance matrix Σ for the SNPs has a block-diagonal form [19, 20]:

(2.1) $$\overset{ˆ}{\Sigma }=\left[\begin{array}{llll} {\overset{ˆ}{\Sigma }}_{1} & & & \\ & {\overset{ˆ}{\Sigma }}_{2} & & \\ & & \ddots & \\ & & & {\overset{ˆ}{\Sigma }}_{b} \end{array}\right]\in R^{p\times p},$$

effectively assuming that SNPs in different blocks are independent. Defining blocks in correspondence to different chromosomes is certainly natural. However, in order to reduce computational burden, researchers typically consider blocks of smaller sizes. It is important to recall that if the block-wise independence reflected in (2.1) is not (at least approximately) satisfied, the knockoff analysis resulting from the pipeline we are about to describe will not have FDR control.

The block boundaries can be computed efficiently using the snp_ldsplit function of the bigsnpr package [20]. Empirically, working in a variety of populations, we found that b on the order of 10^2^ ~ 10^3^ is sufficient to partition the human genome based on the default objective provided in bigsnpr, see Table 1. To obtain reliable estimates to genetic correlation, we recommend that only SNPs with minor allele frequency ≥ 0.01 should contribute to LD block estimation.

**Figure 1:**
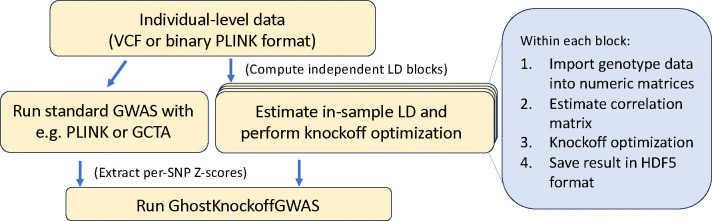
Overview of the GhostKnockoff pipeline. The sequence of events taking place within the solevblock executable are outlined in the blue box.

Within each block i=1,...,b in equation (2.1), solveblock will perform 4 main steps, summarized in Figure 1, and described in the following.

### 2.1 Import genotype data into computer memory

We import the genotype data into double-precision matrices $x_{ij}\in \{0,\;1,\;2,\;missing\}$ using the OpenMendel [21] modules SnpArrays.jl or VCFTools.jl. This step requires $8np_{i}$ bytes of computer RAM, which is memory intensive if $p_{i}$ (number of variants in block i) is extremely large. Typically, however, the block-diagonal approximation (2.1) implies $p_{i}$ is generally on the order of 10^3^, which is simple to handle.

For VCF inputs, our convention is to convert each ALT allele into numeric value of 1. With binary PLINK inputs, all A2 alleles are converted into 1 even if it is the major allele (note this is equivalent to using --keep-allele-order within PLINK 1.9 software). Multi-allelic SNPs should be split prior to analysis. Missing genotypes are automatically imputed by column mean.

### 2.2 Estimation of correlation matrix

Within region ∈{1,…,b}, let $X_{i}\in R^{n\times p_{i}}$ denote the column-standardized genotype matrix. Our next goal is to estimate a correlation matrix ^1)^ ${\overset{ˆ}{\Sigma }}_{i}\in R^{p_{i}\times p_{i}}$ to be used for downstream knockoff optimization. We adopted the same LD estimation routine of Pan-UKB [15] which gave good performance in our previous work [13]. If C denotes a matrix of covariates (sex, age, etc), we compute:

(2.2) $$M_{c}\;=I-C{\left(C^{t}C\right)}^{-1}C^{t}\;(\text{the}\;\text{residual-maker}\;\text{matrix})\;X_{adj}\;=M_{c}X_{i}\;(\text{regressing}\;\text{out}\;\text{effect}\;\text{of}\;\text{covariates})\;{\overset{ˆ}{\Sigma }}_{i}\;=\frac{X_{adj}^{t}X_{adj}}{n}$$

For numerical stability, we set the minimum eigenvalue of ${\overset{ˆ}{\Sigma }}_{i}$ to be 0.00001 and scaled the final result so the diagonal entries are exactly 1. Supplying the matrix of cavariates is made as an optional input in our software.

### 2.3 Parameters for group-knockoffs generation

Our next step is to calculate the parameters that describe the variance-covariance matrix of the original variables and knockoffs. For each block $\Sigma_{i}$, this includes 3 main steps: (1) identify groups of highly correlated SNPs that are going to be the basis of inference, (2) approximate the joint distribution of all SNPs using a conditional independence model in which only a subset of “key variables” within each group are driver of the correlations across group, and (3) find the variance-covariance matrix for $\left(X,\tilde{X}\right)$ that satisfies the exchange-ability constrains while maximizing detection power by maximizing the entropy criteria [22]. The motivation behind all of these steps and the algorithms required to carry them out are described elsewhere [13]. We summarize them below for completeness.

Following [13], for each ${\overset{ˆ}{\Sigma }}_{i}\in R^{p_{i}\times p_{i}}$, we define non-overlapping groups via average-linkage hierarchical clustering with correlation cutoff 0.5. Given group boundaries, we then apply the best subset selection algorithm of [13] (Algorithm A2 in the supplement) with c=0.5 to identify *group-key* variables within each group. Intuitively, the group-key variables are a subset of $\left\{1,\ldots ,p_{i}\right\}$ that drive the dependence across groups. Let R denote the set of group-key variables, C denote its complement, and ${\left({\overset{ˆ}{\Sigma }}_{i}\right)}_{\left(R,R\right)}$ the rows and columns of ${\overset{ˆ}{\Sigma }}_{i}$ corresponding to variables in R. Knockoff optimization proceeds by solving the maximum entropy [22] objective

(2.3) $$\underset{S_{i}}{max}\log det\left(\frac{m\;+\;1}{m}{\left({\overset{ˆ}{\Sigma }}_{i}\right)}_{\left(R,R\right)}-S_{i}\right)+m\;log\;det\left(S_{i}\right)\;\text{subject}\;\text{to}\;\left\{\begin{array}{l} \frac{m\;+\;1}{m}{\left({\overset{ˆ}{\Sigma }}_{i}\right)}_{\left(R,R\right)}-S_{i}⪰0\\ S_{i}⪰0 \end{array}\right.$$

where m=5 is the number of knockoffs to generate per feature and $S_{i}⪰0$ means $S_{i}$ is positive semi-definite. Then the $p_{i}\times p_{i}$ matrix

$$D_{i}\;=\left(\begin{array}{cc} S_{i} & S_{i}{\left({\overset{ˆ}{\Sigma }}_{i}\right)}_{\left(R,R\right)}^{-1}{\left({\overset{ˆ}{\Sigma }}_{i}\right)}_{\left(R,C\right)}\\ {\left({\overset{ˆ}{\Sigma }}_{i}\right)}_{\left(C,R\right)}{\left({\overset{ˆ}{\Sigma }}_{i}\right)}_{\left(R,R\right)}^{-1}S_{i} & D_{i,22} \end{array}\right)\;\text{where}\;D_{i,22}\;={\left({\overset{ˆ}{\Sigma }}_{i}\right)}_{\left(C,C\right)}-{\left({\overset{ˆ}{\Sigma }}_{i}\right)}_{\left(C,R\right)}{\left({\overset{ˆ}{\Sigma }}_{i}\right)}_{\left(R,R\right)}^{-1}{\left({\overset{ˆ}{\Sigma }}_{i}\right)}_{\left(R,C\right)}+{\left({\overset{ˆ}{\Sigma }}_{i}\right)}_{\left(C,R\right)}{\left({\overset{ˆ}{\Sigma }}_{i}\right)}_{\left(R,R\right)}^{-1}S_{i}{\left({\overset{ˆ}{\Sigma }}_{i}\right)}_{\left(R,R\right)}^{-1}{\left({\overset{ˆ}{\Sigma }}_{i}\right)}_{\left(R,C\right)}$$

is the solution to the maximum entropy objective for the full ${\overset{ˆ}{\Sigma }}_{i}\in R^{p_{i}\times p_{i}}$. In particular, $D_{i}⪰0$ satisfies the constraints $\frac{m+1}{m}{\overset{ˆ}{\Sigma }}_{i}-D_{i}⪰0$ and can be used for ghost-knockoff sampling [23]. We used the coordinate descent algorithm implemented in Knockoffs.jl [13] to solve equation (2.3). Note that this procedure implicitly makes the second order assumption that the genotypes $\left(X_{1},\ldots ,X_{p_{i}}\right)∼𝒩(0,{\overset{ˆ}{\Sigma }}_{i})$ [24].

### 2.4 Output in HDF5 format

Up until this point, we have assembled the reduced data:

- ${\overset{ˆ}{\Sigma }}_{1},\ldots ,{\overset{ˆ}{\Sigma }}_{b}:$ correlation matrices for each block
- $S_{1},\ldots ,S_{b}:$ knockoff optimization matrices for each block, carried out on the group-key variables
- $D_{1},\ldots ,D_{b}:$ knockoff sampling matrices for each block satisfying $\frac{m+1}{m}{\overset{ˆ}{\Sigma }}_{i}-D_{i}⪰0$.
- Group membership vector for each block i={1,...,b}
- Group-key variables for each block i={1,...,b}

We will save this ensemble of data in HDF5 format [25] which can be directly read by GhostKnockoffGWAS [12]. Note that due to the block-diagonal partitioning in equation (2.1), this bundle of “pre-processed" LD files are typically dozens of gigabytes in size, and contain no individual level data. We emphatise this last point to invite researchers to share their results as they may be used by other studies on similar populations.

## 3 Result

To illustrate the software, we are going to leverage simulations and real data analysis. Both are based on data obtained from the UK-Biobank. We start by describing the data we use and its pre-processing steps.

### 3.1 UK-Biobank (UKB) data

#### Quality Control.

The second release of the UK Biobank (UKB) data [14] contains ~ 500,000 samples of primarily European descent and ~ 800,000 SNPs without imputation. Following [3], for British samples, we kept only 1 sample from each of the 60,169 familial groups. This resulted in *n* = 306,604 unrelated British samples. Next, we also created different sub-populations based on self-reported Indian (*n* = 5951), Caribbean (*n* = 4517), African (*n* = 3394), and Chinese (*n* = 1574) ancestry. For these non-British sub-populations, we kept related samples to mimic cryptic relatedness in downstream simulations. For all five UKB subpopulations, we excluded non-biallelic SNPs with minor allele frequency (MAF) < 0.01 and Hardy-Weinburg equilibrium p-values < 10^−6^. Finally, for our real-data analysis, we chose 26 continuous and commonly measured phenotypes (see Table 2) as targets of analyses. Samples that cannot be matched to phenotype file by ID or whose phenotypes contain missing values were also excluded.

#### Non-genetic covariates.

Following Pan-UKB [15], we included the following covariates for LD adjustments: sex, age, age^2^, sex×age, sex×age^2^, and the top 10 principal components precomputed by the UK-Biobank. These covariates form the matrix C in equation (2.2).

### 3.2 LD file Construction

Our first goal is to construct the necessary LD files for various subpopulations of the UKB, to enable knockoff-based analysis and simulations on these populations. To do so, we first applied the snp_ldsplit functionality of bigsnpr package [20] to each chromosome of each subpopulation of UKB separately. We used the parameters thr_r2 = 0.01, max_r2 = 0.3, min_size = 500, and max_size = {1000, 1500, 3000, 6000, 10000}. Given block boundaries in each subpopulation, we applied our solveblock executable directly on the individual-level genotypes. We submitted a separate cluster job for each region, and every job finished within a few minutes. Table 1 summarizes the final number of blocks and disk space needed to store the resulting LD files.

### 3.3 Simulations: FDR control

In this section, we demonstrate the proposed pipeline results in valid (group) FDR control when the output of solveblock is directly used by GhostKnockoffGWAS [12]. We restrict our attention to SNPs falling on chromosome 22, totaling p=8878 SNPs. In all simulations we column-standardize X and simulate the response as $y=X\beta +𝒩\left(0,3I_{n\times n}\right)$ where β contain k=10 non-zero effects chosen randomly and effect sizes drawn from 𝒩(0,0.5). The narrow sense heritability for these simulations is approximately 0.25. After regressing out the effect of covariates, we standardize the phenotype.

Marginal Z-scores were computed in two ways. For the British samples, where related individuals are removed, we compute marginal Z-scores by the score statistic $z=X^{t}y/\sqrt{n}$ where y is standardized. For other populations in the UKB, which contain related samples and potential continental admixture, we use the linear mixed model software GCTA [26] to compute p-values, and transformed them back into Z-scores. Once LD files are estimated from genotypes, and Z-scores evaluated, they are used as the only inputs used for GhostKnockoffGWAS for evaluating power and (group) FDR.

**Figure 2:**
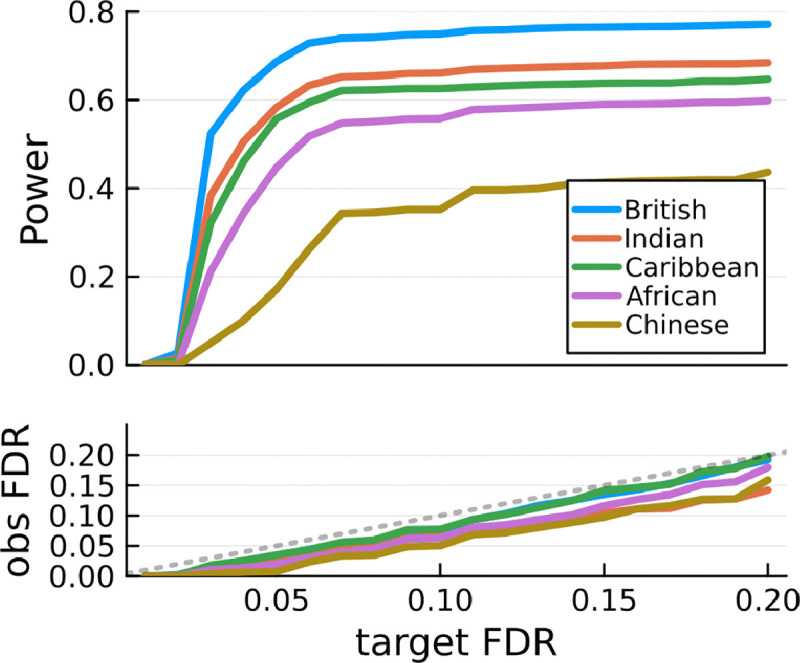
Power and FDR simulation study using UKB. The British subset contains only unrelated individuals. For other populations, related samples are included in the analysis.

We repeat each simulation 200 times, and the result is depicted in Figure 2. As expected, the LD files from solveblock can be used with GhostKnockoffGWAS to control (group) FDR in analyzing GWAS summary statistics data. We can also directly compare power across all populations since the effect sizes in all simulations are generated from the same distribution, which makes it clear that power increases as a function of sample size.

### 3.4 Real data analysis: 26 UKB phenotypes using GhostKnockoff

Here we analyzed 26 continuous phenotypes based on the British samples of UKB. The goal of this section is to (1) demonstrate the scalability of GhostKnockoff on biobank-scale datasets (each phenotype took <15 minutes to run on a single CPU), and (2) demonstrate the enhanced power of GhostKnockoff in locus discovery compared to marginal analysis methods.

Because GhostKnockoffGWAS relies on marginal Z-scores as inputs, we first conduct a standard marginal analysis using the score statistic after regressing out the effect of covariates from the phenotype vector. Mathematically, let y be the original phenotype vector, C be the matrix of covariates, and X the genotype matrix that is column standardized to mean 0 variance 1. We first compute $\tilde{y}=(I-C{\left(C^{t}C\right)}^{-1}C^{t})y$ and standardize it to mean 0 variance 1. Then we compute $z=X^{t}\tilde{y}/\sqrt{n}$ where n=306,629 British samples. The resulting z in combination with the output of solveblock are then used as inputs for GhostKnockoffGWAS [12].

**Figure 3:**
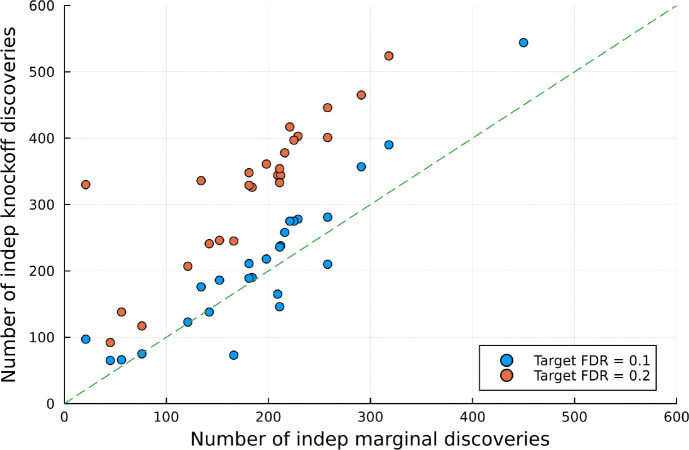
The number of (independent) discoveries from GhostKnockoff compared to a conventional marginal GWAS. Each dot is a different phenotype (26 total), each of which took <15 minutes on a single CPU to run. Dots above the dashed line indicate that knockoff analysis discovered more signals compared to marginal analysis. A table formatted result is available in Table 2.

Figure 3 compares the number of independent discoveries from knockoff-based analyses to that from conventional marginal GWAS. For both the marginal and ghost-knockoff analysis, 2 discoveries are considered *independent* if they are at least 1 Mb apart. Based on this criteria, ghost knockoff analyses found 19.0 (149.1) additional independent discoveries compared to marginal testing when the target FDR is 10% (20%). A table formatted result is available in Table 2. Notably, ghost-knockoff discovered more signals in 20 out of 26 traits given target FDR of 10%, and each phenotype took 15 minutes or less to run on a single CPU core on the cloud. These result showcases our method’s enhanced power and computational efficiency for locus discovery.

## 4 Discussion

The goal of this paper is two fold. First, we introduce the solveblock executable. When combined with the accompanying software GhostKnockoffGWAS [12], one can perform an end-to-end GWAS analysis of individual-level data from the ghost-knockoff perspective by running a standard GWAS followed by a ghost-knockoff analysis. We found that this approach is scalable to biobank-sized data, controls the FDR in extensive simulations, while delivering enhanced power for locus discovery compared to traditional marginal association testing methods.

The second goal of this paper is to provide a platform for data exchange and to encourage the procedure. Before this paper, LD panels necessary for GhostKnockoff analyses are limited to European samples. Here we additionally provide LD files for Indian, Caribbean, African, and Chinese samples. In addition, the solveblock executable introduced in this paper will allow investigators to generate their own LD panels. By construction, these LD files are small in size and can be shared freely. If just a small fraction of our users are open to data-sharing, GhostKnockoffGWAS will become exponentially more versatile. For example, one can upload the result to Zenodo [16] and share the link with us, which we will then post to the GhostKnockoff documentation page with an appropriate reference. Although such a resource is currently part dream part reality, it could be achieved by a concerted international effort.

Several lines of research are planned or ongoing. First, similar to Michigan’s imputation server [27], it is feasible for all ghost-knockoff computation to happen entirely on the cloud due to its computational simplicity. With such an ideal resource, users upload GWAS summary statistics, select the desired LD panel to use, and later download the ghost-knockoff discoveries. This bypasses the need to distribute processed LD files and avoids headaches with software installation. Next, it is now realistic to apply the solveblock executable to other datasets, such as the All of US [28] and Million Veteran Project [29]. Enabling GhostKnockoff analyses on these newer biobanks will potentially increase the power of thousands of related projects, but also improve health disparities for minorities who are not well represented in the UK Biobank.

Finally, it is of particular interest to us to extend the ghost-knockoff methodology to non-SNP-array data, such as those generated from high-coverage whole genome sequencing or low-pass imputation-based data [30]. Such dense genotyping technologies are expected to become increasingly affordable, eventually leading to a higher adoption rate than array-based data in genetic studies. While the simulation studies here show empirical FDR control, the current GhostKnockoff pipeline is likely too slow for analyzing high-coverage sequenced data, since the blocks in equation (2.1) routinely have dimensions exceeding 10^5^. In such cases, more computational innovation is needed for knockoff optimization (2.3) and GhostKnockoff sampling [12]. We raise these research questions to stimulate discussions and encourage participation from the broader community.

## Figures and Tables

**Table 1:** Summary of various LD files after applying the snp_ldsplit function of bigsnpr package to the individual-level data of UK-Biobank.

Data	Sample size (*n*)	Number of blocks (b)	disk space (GB)

British	306604	636	20.0
Indian	5951	615	13.0
Caribbean	4517	489	13.0
African	3394	513	9.4
Chinese	1574	505	7.4

**Table 2:** The number of independent discoveries for various phenotypes analyzed under both the knockoff framework and the conventional marginal hypothesis testing.

Phenotype	# marginal	GK FDR 10%	GK FDR 20%	GK runtime (seconds)

Height	450	544	722	533
Weight	258	210	401	540
Waist circumference	166	73	245	540
Body mass index	209	165	344	760
Body fat mass	211	146	333	485
Diastolic blood pressure	121	123	207	746
Systolic blood pressure	142	138	241	698
Whitecell	184	190	326	756
Redcell	258	281	446	766
Haemoglobin	198	218	361	768
Haematocrit	181	211	348	717
Corpuscular volume	229	278	403	754
Corpuscular haemoglobin	212	238	344	835
Redcell width	152	186	246	759
Platelet	318	390	524	540
Platelet volume	291	357	465	805
Platelet width	216	258	378	693
Lymphocyte	56	66	138	711
Monocyte	134	176	336	705
Neutrophill	211	236	354	824
Eosinophill	181	189	329	725
Basophill	45	65	92	700
Nucleated redcell	21	97	330	484
Reticulocyte	76	75	330	485
Reticulocyte volume	225	275	397	485
Spheredcell volume	221	275	417	492

GK stands for ghost-knockoff and FDR stands for false-discovery rate. The target FDR is set to 10% and 20%. Note the runtime corresponds to the runtime of GhostKnockoffGWAS and does not include the runtime for solveblock, since the later only needs to be done once for a population of interest.

## Footnotes

1) Note that in the pre-processing step of identifying block boundaries, there is technically another correlation matrix implicitly used by snp_ldsplit function of bigsnpr package. That matrix is only used to identify block boundaries. Once the boundaries are identified, the correlation matrix that we estimate (denoted by ${\overset{ˆ}{\Sigma }}_{i}$) are what is actually used in the GhostKnockoff pipeline.
